# Chitosan Treatment Delays the Induction of Senescence in Human Foreskin Fibroblast Strains

**DOI:** 10.1371/journal.pone.0140747

**Published:** 2015-10-14

**Authors:** Ching-Wen Tsai, Yu-Ting Kao, I-Ni Chiang, Jyh-Horng Wang, Tai-Horng Young

**Affiliations:** 1 Institute of Biomedical Engineering, College of Medicine and College of Engineering, National Taiwan University, Taipei 100, Taiwan, No.1, Sec. 1, Jen - Ai Rd., Taipei 100, Taiwan; 2 Department of Urology, National Taiwan University Hospital, Taipei 100, Taiwan, No.7, Chung-Shan S. Rd., Taipei 100, Taiwan; 3 Department of Orthopedic Surgery, National Taiwan University Hospital, Taipei 100, Taiwan, No.7, Chung-Shan S. Rd., Taipei 100, Taiwan; University of Newcastle, UNITED KINGDOM

## Abstract

Fibroblasts have been extensively used as a model to study cellular senescence. The purpose of this study was to investigate whether the human foreskin fibroblast aging process could be regulated by using the biomaterial chitosan. Fibroblasts cultured on commercial tissue culture polystyrene (TCPS) entered senescence after 55–60 population doublings (PDs), and were accompanied by larger cell shape, higher senescence-associated β-galactosidase (SA β-gal) activity, lower proliferation capacity, and upregulation of senescence-associated molecular markers p21, p53, retinoblastoma (pRB), and p16. Before senescence was reached, PD48 cells were collected from TCPS and seeded on chitosan for three days (PD48-Cd3) to form multicellular spheroids. The protein expression of senescence-associated secretory phenotypes (SASPs) and senescence-associated molecular markers of these cells in PD48-Cd3 spheroids were downregulated significantly. Following chitosan treatment, fibroblasts reseeded on TCPS showed lower SA β-gal activity, increased cellular motility, and a higher proliferation ability of 70–75 PDs. These phenotypic changes were not accompanied by colonies forming in soft agar and a continuous decrease in the senescence-associated proteins p53 and pRB which act as a barrier to tumorigenesis. These results demonstrate that chitosan treatment could delay the induction of senescence which may be useful and safe for future tissue engineering applications.

## Introduction

In general, primary cells *in vitro* cannot proliferate indefinitely due to replicative senescence [1]. Important features of replicative senescence include cell enlargement and flattening [2–6], enhanced specific senescence-associated β-galactosidase (SA β-gal) activity [7–9] and upregulation of cell-cycle related gene p53 and its downstream gene p21 [10] or p16-pRB pathway [11,12]. Recent studies have found that some proinflammatory cytokines, chemokines, and protease are secreted from senescent cells, called senescence-associated secretory phenotypes (SASPs). SASPs would alter tissue microenvironment, attract immune cells, and induce aging-related inflammation and diseases [13,14]. Once cells become irreversibly arrested, cells cannot divide again by the stimulation of growth factors [15]. Therefore, the senescence response is regarded as a barrier to tumorigenesis [16,17].

Recently, many chemicals and techniques have been tested to induce or delay cellular senescence. For example, hydrogen peroxide [18], doxorubicin [19–21], etoposide [22–24], cilostazol [25,26], staurosporine [27,28], and beryllium [29] can induce premature senescence. In addition, the introduction of Ras oncogenes is capable of inducing the senescence response in young cells [30]. On the other hand, regulation of extracellular matrix [31], oxygen level [32–34] or serum concentration [35] has been used to enhance proliferation to delay cellular senescence. These findings indicate cellular senescence process is potentially regulated by appropriate methods. Similarly, cells can respond to the underlying biomaterial, so it is possible that the cellular senescence program might be influenced by the characteristic properties of the biomaterial. However, to our knowledge, cellular senescence determined by biomaterials and the signaling events involved are poorly understood [36].

Chitosan is a unique polysaccharide, modified from chitin with biodegradable, biocompatible, and pH-sensitive properties [37–40]. These advantages make chitosan more accessible for tissue engineering applications. Chitosan is widely used in cosmetic products of skin [41,42] and treatment of aging-related diseases [43] but its utility in enhancing cellular proliferation, migration and lifespan has seldom been addressed. In this study, we tested whether human foreskin fibroblast aging process could be regulated by using chitosan as the cell substrate. We hope cells can be maintained in a more healthy state for further biomedical applications.

## Materials and Methods

### Preparation of chitosan substrates

Chitosan (Sigma, C-3646) solution (1%) in 0.5M acetic acid (Sigma) was coated on 6-well TCPS (Corning) and dried at 60°C overnight to form a thin film [44]. Then, the plates were neutralized by 0.5N NaOH (Sigma), washed thoroughly with water, and exposed to ultraviolet light for 30 minutes for subsequent cell culture.

### Cell culture

Human foreskin fibroblasts were isolated from specimens of donors who needed to undergo circumcision. Six strains were provided in this study and the ages of donors were about fifteen to thirty. Donors provide their written consent to participate in this study. The study protocol was approved by the Institutional Review Board of the National Taiwan University Hospital. The culture method has been described elsewhere [45]. Briefly, after removing the subcutaneous tissue and the epidermal layer of the specimens, fibroblasts were obtained from the dermal layer by the explant outgrowth technique with dispase (Roche) treatment. These cells were then maintained in Dulbecco's Modified Eagle Medium (DMEM, Gibco), which contained 10% fetal bovine serum (FBS, Biological Industries), 100 U/ml penicillin G, 100 μg/ml streptomycin, 250 μg/ml amphotericin B (Biological Industries) at 37°C in a humidified chamber with 5% CO_2_ and 95% air atmosphere.

Cells were routinely seeded in 6-well TCPS at the density about 5000/cm^2^ and were subcultured and counted every 5 days. For chitosan treatment, cells were harvested from TCPS with trypsin/ Ethylenediaminetetraacetic acid (trypsin/EDTA, Gibco) and seeded on chitosan-coated surfaces at about 25000/cm^2^ for 3 and 7 days. Cell morphology was visualized under a microscope (Olympus IX71). The population doubling times (PDs) was calculated as follows: PDs = log_2_ (number of cell harvest at subculture/number of cells initially seeded).

### Senescence-associated β-galactosidase (SA-β-gal) staining

Cells were fixed in 4% formaldehyde and incubated with fresh SA β-gal staining solution at pH 6 in the absence of CO_2_ at 37°C [7,9,46]. The stained cells with blue sedimentation in the cytoplasm were photographed and the percentages of stained cells were counted.

### Viability assay

Cell viability in the multicellular spheroids was observed by the Live/Dead kit (Invitrogen, 3224). Live cells stained with calcein AM (green) and dead cells labeled with ethidium homodimer (red) were observed under a confocal fluorescence microscope (TCS SP5, LEICA). Moreover, fibroblast spheroids were trypsinized, dissociated and stained with the Alexa Fluor^®^ 488 Annexin V/Dead Cell Apoptosis Kit (Invitrogen, V13241) according to the manufacture’s protocol. The cell viability was quantified by flow cytometry on a FACSCalibur flow cytometer (BD Biosciences).

### Western blotting

Cells were lysed with commercial lysis buffer (RIPA, Roche Diagnostics GmbH). Proteins from lysed cells were separated by sodium dodecyl sulfate polyacrylamide gel electrophoresis (SDS-PAGE) and then transferred onto nitrocellulose membranes. After blocking in bovine serum albumin, the membranes were probed with human primary antibodies against p53 (Merck OP09; PAb1891; 1:200), p21 (Cell Signaling #2946; DSC60; 1:2000), p16 (BD Pharmingen 551153; G175-405; 1:1000), pRB (Merck OP66; LM95.1; 1:100), TGF-β (Cell signaling 3709; 56E4; 1:1000), IL-1β (Santa cruz sc-7884; H-153; 1:1000), IL-6 (GeneTex GTX110527, polyclone; 1:1000), IL-8 (GeneTex GTX115959; polyclone; 1:1000), GAPDH (Abcam ab22555; polyclone; 1:10000), incubated in HRP-conjugated secondary antibodies (Goat anti-rabbit IgG (Abcam ab97051; 1:5000) and Goat anti-mouse IgG (Abcam ab97023; 1:5000)) and visualized by enhanced chemiluminescence (ECL, Millipore).

### BrdU assay

Cells were incubated with BrdU (5-bromo-2’-deoxiuridine) for 24 hours and then BrdU incorporation during DNA synthesis in replicating cells was assayed according to the protocol of the Cell Proliferation ELISA, BrdU kit (Roche, 11 647 229 001). Results were quantified using an ELISA reader (Spectra Max M2e, Molecular Devices) at dual wavelength of 450–595 nm. Before incorporated with BrdU, cells, treated with 1.5mM H_2_O_2_, were H_2_O_2_-treated group.

### Cell cycle analysis

Cells were collected and fixed with 80% ethanol solution overnight at -20°C. After removing ethanol, cells were stained with 50 μg/mL propidium iodide (PI) solution containing 0.1% (v/v) Triton X-100, and 100 μg/mL DNase-free RNase A for 30 minutes, and analyzed using flow cytometry.

### Scratch assay

Cells were cultured in 24-well TCPS at the density of 20,000/cm^2^ for five days to reach confluence. The well surface was gently scratched with a pipette tip to create a rectangular scratch. After removal of culture medium and detached cells, remaining cells were washed twice with PBS and cultured in DMEM with 10% FBS. The cell images were photographed under Leica DMI600 microscope, and closure of the wound was analyzed using ImageJ software.

### Soft agar assay

The base gel was prepared by mixing a 1% agar solution and 2X DMEM with 20% FBS. The top gel consisted of 0.35% agarose solution, DMEM with 10% FBS and cells. Cells were cultured in soft agar for 2 weeks and colony formation was visualized by microscopy.

### Cell sorting with the size and auto-fluorescence

Cells were detached and sorted according to the size and auto-fluorescence by flowcytometry. Cells expressing both forward-scattered light (FSC) and fluorescein isothiocyanate (FITC) were considered the older population, and those without the expression were considered the younger population.

### Statistical analysis

All measurements were shown as the mean ± standard deviation (SD) from at least three independent experiments. Statistical significance was determined using one-way analysis of variance (ANOVA) followed by Duncan’s test. P value of < 0.05 and 0.01 was considered significant.

## Results

### Senescent-like changes in human foreskin fibroblasts

**Fig 1:**
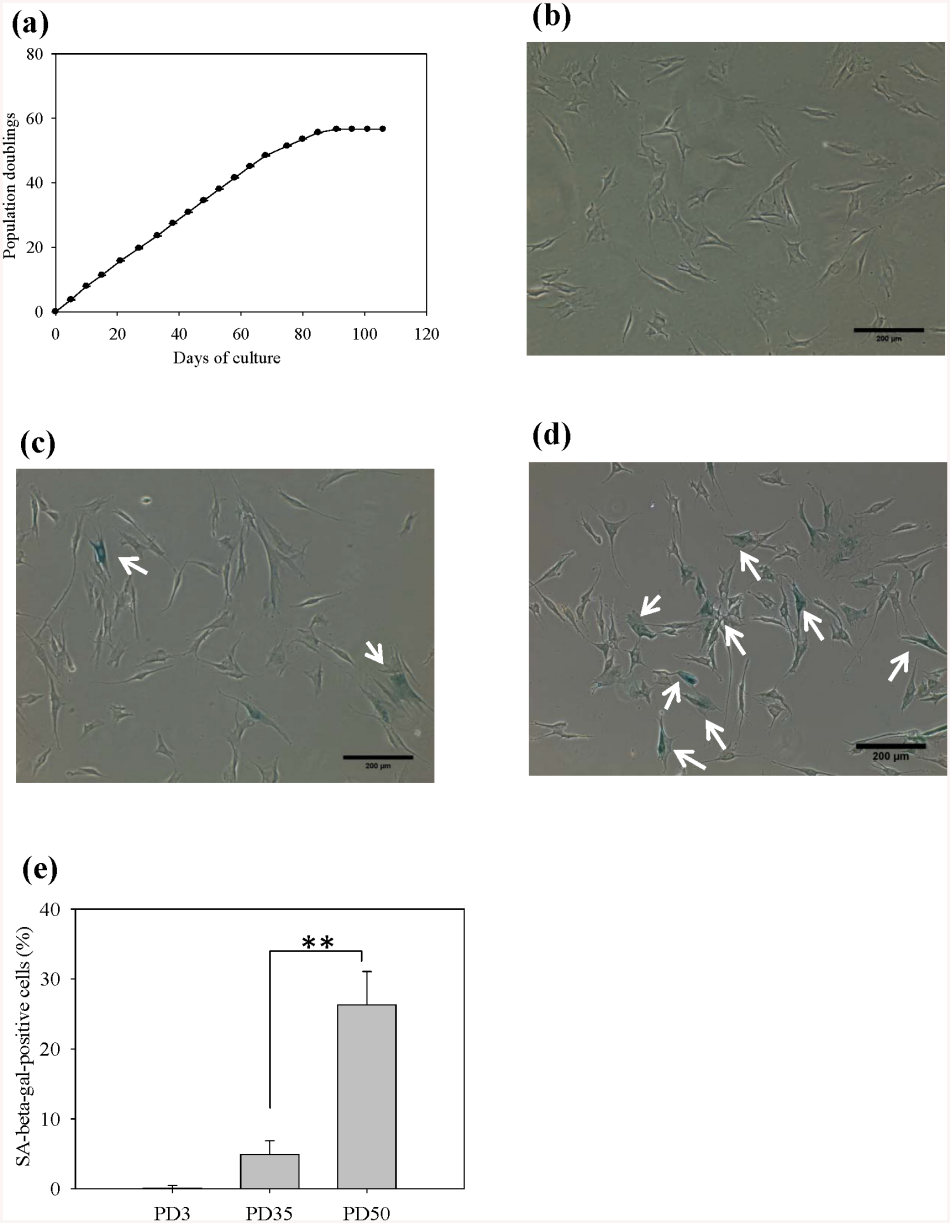
Fibroblasts were serially passaged on TCPS. (a) Population doublings (PDs) were determined after each passage and plotted against days in culture (n = 3). SA-β-gal staining of fibroblasts at (b) PD3, (c) PD35, (d) PD50. Scale bar = 200 μm. (e) The percentage of cells stained for SA-β-gal. At least 400 cells were calculated from ten randomly selected fields for each case. **p<0.01.

To test whether the senescent phenotype of fibroblasts could be regulated by chitosan, human foreskin fibroblasts were firstly incubated on TCPS to measure the accumulated population doublings (PDs). Fig 1a shows fibroblasts ceased dividing when they proliferated to approximately PD55-60. Cell morphology at PD3, 35 and 50 were shown in Fig 1b–1d. Clearly, more cells with enlarged morphology which is a typical senescent feature were observed at PD50 compared to PD3. In addition to cell growth and morphology evaluation, cells were stained by SA-β-gal, an important senescence biomarker [46]. As expected, cells with enlarged morphology exhibited an obvious SA β-gal staining (Fig 1b–1d). Fig 1e shows the percentage of SA β-gal-positive cells increased with increasing PD numbers. Almost no cells were positively stained at an early passage of PD3. Compared to PD35 cultures, the percentage of cells staining positive for SA β-gal at PD50 cultures was significantly increased (p<0.01). These results were consistent with the concept that fibroblasts having divided many times would be closer to cellular senescence.

### The effect of chitosan treatment on delaying cellular senescence

**Fig 2:**
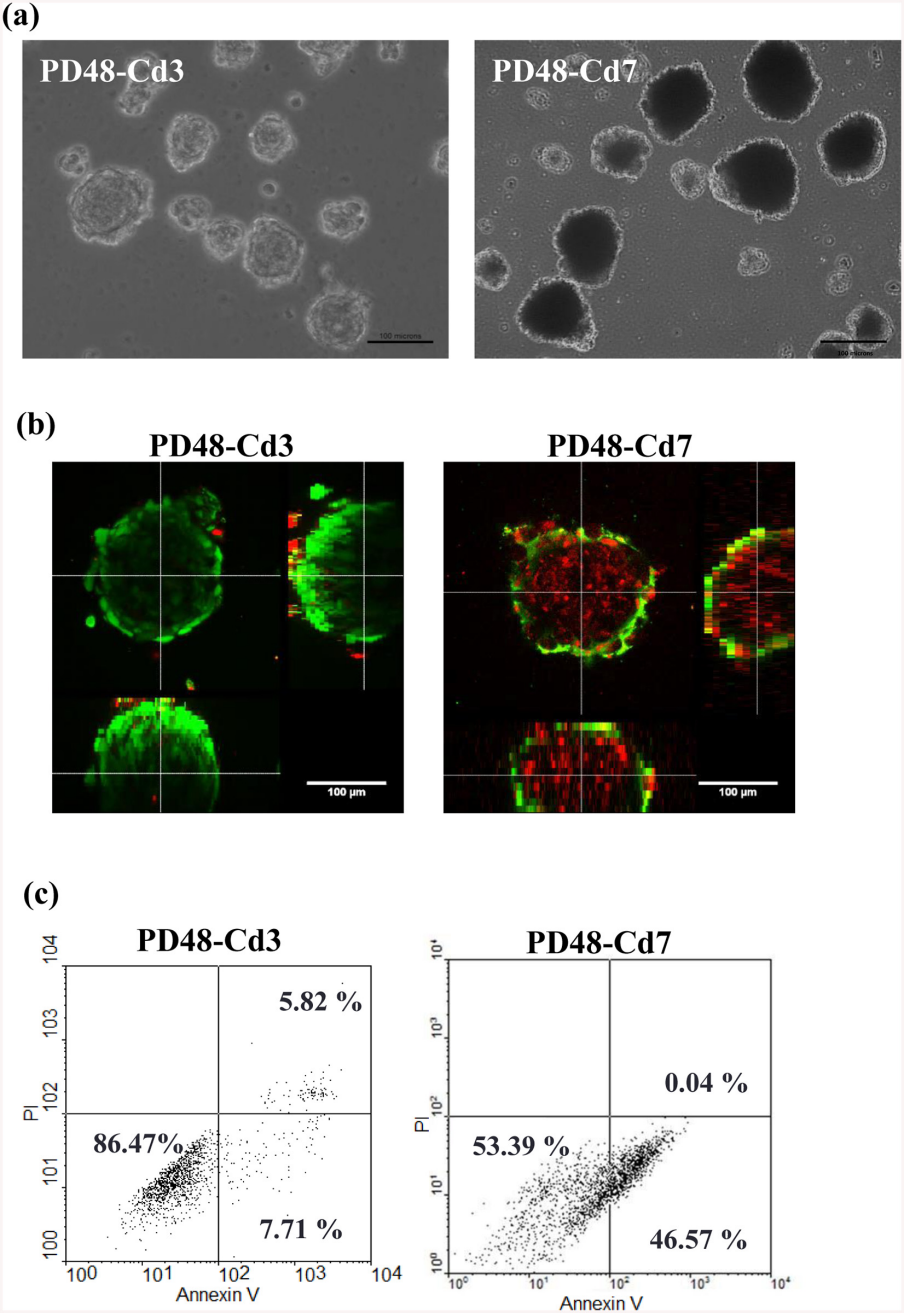
PD48 fibroblasts cultured on chitosan for 3 days (PD48-Cd3) and 7 days (PD48-Cd7) to form multicellular spheroids. (a) Optical image of multicellular spheroids. (b) Confocal microscopic image of live/dead cells. Live cells (green) were stained with calcein AM. Dead cells (red) were stained with ethidium homodimer. (c) Viability of cells within the multicellular spheroids determined by PI/annexin-V-FITC labeling. Scale bar = 100 μm.

Before senescence (about PD55) was reached, PD48 cells were collected and seeded on chitosan. After 3 days (PD48-Cd3) and 7 days (PD48-Cd7) of culture, fibroblasts suspended above chitosan to form multicellular spheroids with the size about 50–100 μm (Fig 2a). Based on the live/dead assay, the cells of the majority in PD48-Cd3 spheroids were viable, but PD48-Cd7 spheroids were almost stained ethidium homodimer (Fig 2b). Flow cytometry assay showed the percentages of viable cells within the PD48-Cd3 spheroids were more than 85%, indicating no accelerated apoptosis and necrosis for cells cultured on chitosan for 3 days (Fig 2c). With increasing chitosan treatment time, the percentage of viable cells within the PD48-Cd7 spheroids decreased (53.39%). Therefore, we chose 3 days of culture above chitosan for the subsequent senescence tests.

**Fig 3:**
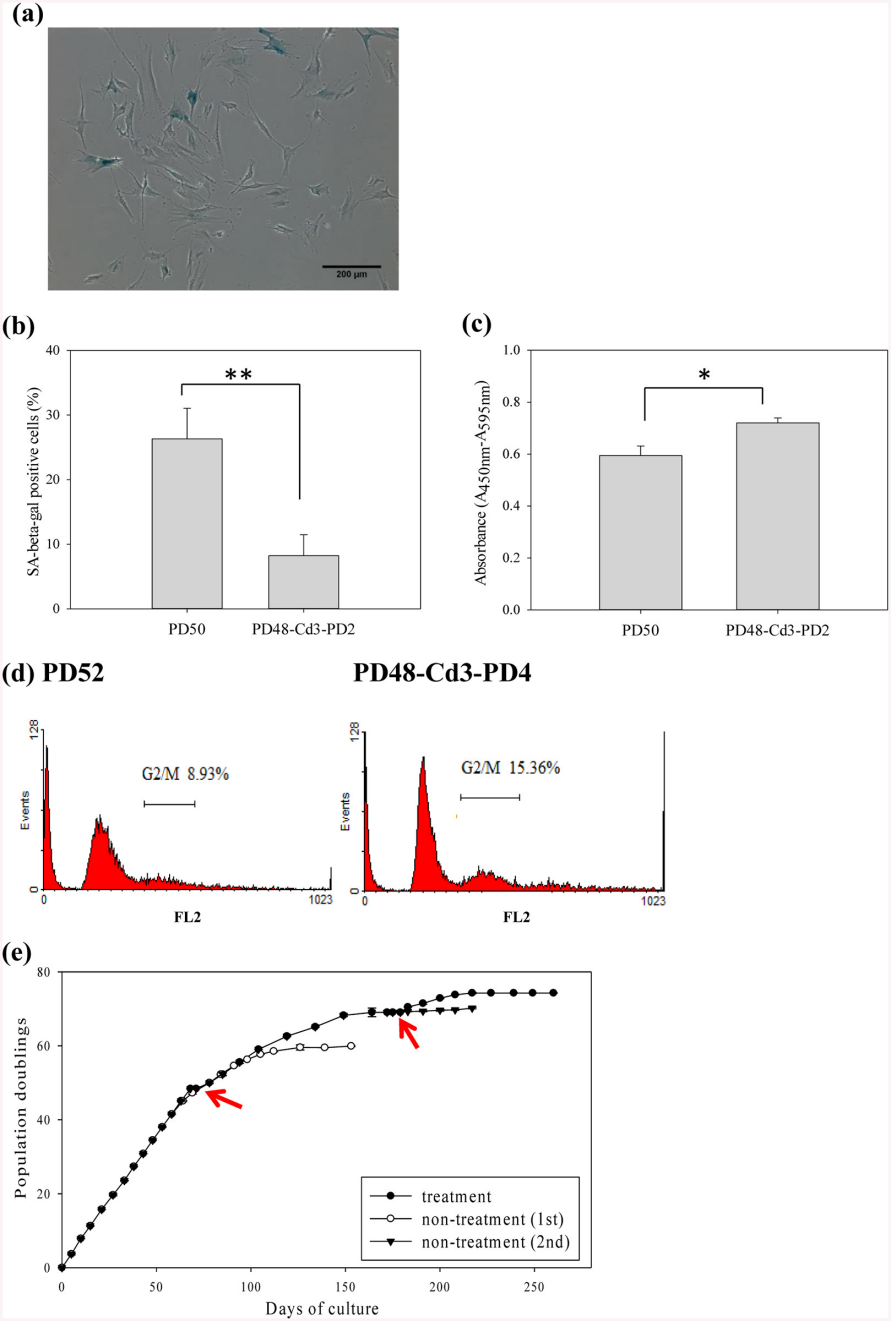
Fibroblast spheroids were reseeded on TCPS after chitosan treatment. (a) SA-β-gal staining of PD48-Cd3-PD2 cells. Scale bar = 200 μm (b) The percentage of PD50 and PD48-Cd3-PD2 cells stained for SA-β-gal. At least 400 cells were calculated from ten randomly selected fields for each case. **p<0.01. (c) BrdU incorporation assay of PD50 and PD48-Cd3-PD2 cells (n = 4). *p<0.05. (d) Cell cycle (PI staining) analysis of PD52 and PD48-Cd3-PD2 cells. (e) The PD curve with and without chitosan treatment (n = 3). Red arrows indicate PD48 (1st treatment) and PD70 (2nd treatment) cells were seeded on chitosan for 3 days and reseeded on TCPS for serial passages.

Firstly, PD48-Cd3 spheroids were collected from chitosan surface and reseeded on TCPS for serial passages. Cell spheroids attached onto the TCPS surface and cells migrated out of spheroids. Fig 3a shows most chitosan treated PD48 cells after reseeded on TCPS for 2 additional doublings (PD48-Cd3-PD2) grew into thin and long morphology. Compared to PD50 cell, PD48-Cd3-PD2 cells could significantly decrease the percentage of SA β-gal-positive cells (p<0.01) from more than 20% to less than 10% (Fig 3b). On the other hand, to investigate whether chitosan treatment increased fibroblast PD numbers by increasing proliferation, BrdU cell proliferation assay and PI staining were used to examine cell proliferation capacity in prepared cells. BrdU incorporation assay revealed significant increase in proliferation of chitosan-treated PD48-Cd3-PD2 cells compared to PD50 cells (p<0.05, Fig 3c). Similarly, the ratio of the G2/M phase in chitosan-treated cells was increased (Fig 3d). Interestingly, Fig 3e shows PD48 cells after chitosan treatment were able to undergo approximately 20 additional doublings on TCPS. Furthermore, PD70 cells were treated with chitosan again for 3 days of culture. The second treatment by chitosan could extend the number of PD to 75 (Fig 3e).

### Effect of chitosan treatment on cell migration

**Fig 4:**
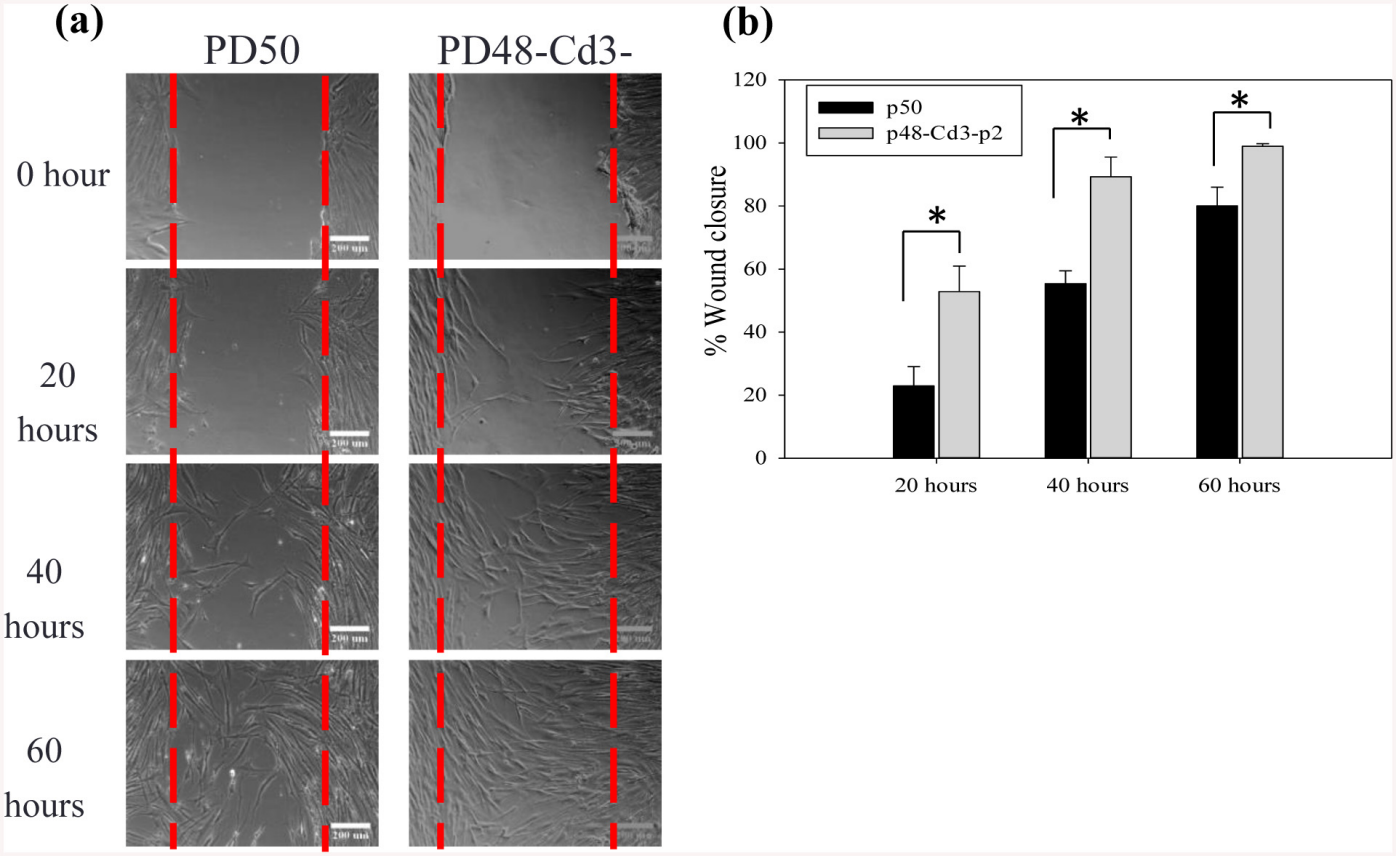
Effect of chitosan treatment on scratch wound assay. (a) Both wounds were gradually filled with PD50 and PD48-Cd3-PD2 cells. (b) The percentage of wound closure by PD50 and PD48-Cd3-PD2 cells (n = 5). **p<0.01.

The migration ability of chitosan-treated and untreated cells was assessed using a scratch wound model. Fig 4 shows the wound was more rapidly filled by PD48-Cd3-PD2 cells than by PD50 cells, confirming the migratory ability of chitosan-treated cells. Quantification analysis showed that the wound of PD48-Cd3-PD2 cells was completely repaired after 60 hours and exhibited a significant difference compared to PD50 cells (p<0.01).

**Fig 5:**
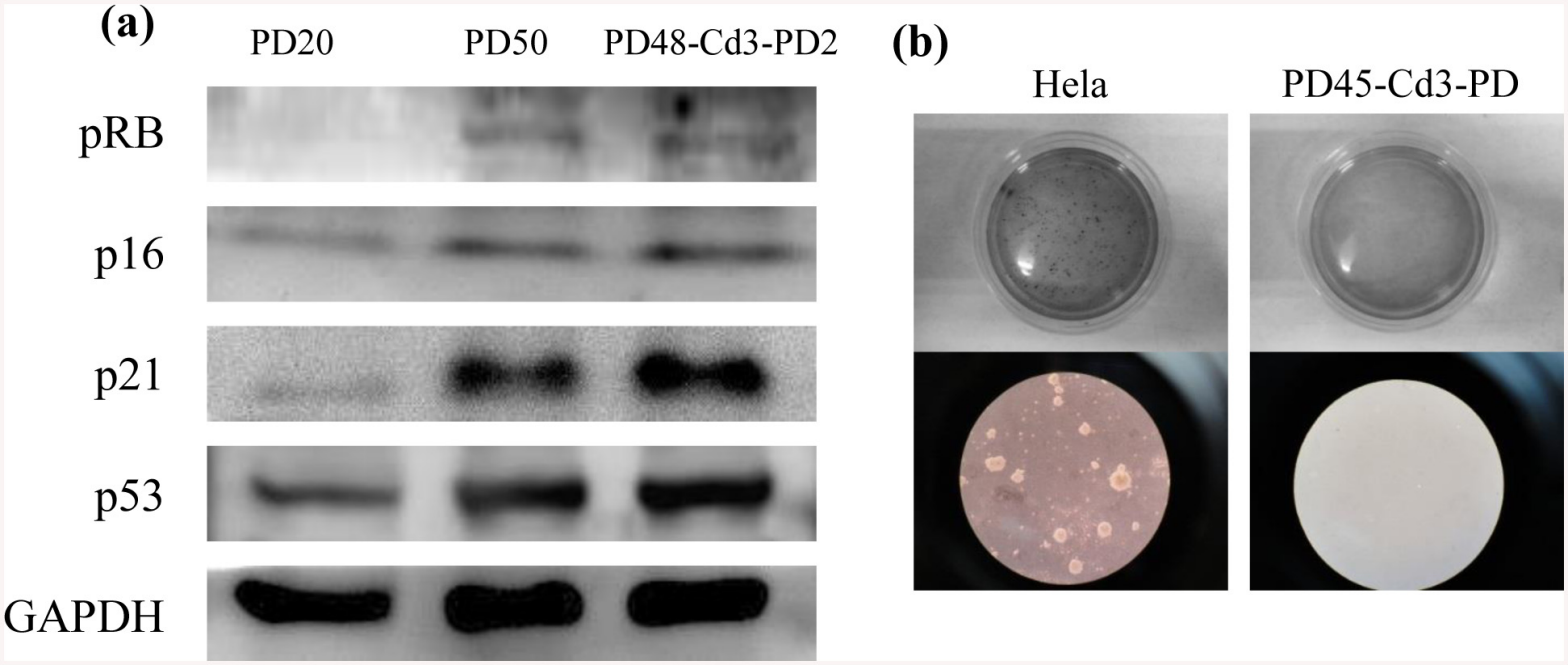
The characteristics of fibroblasts after chitosan treatment. (a) Western blot results of senescence-associated protein pRB, p16, p21 and p53 expression in PD20, PD50, and PD48-Cd3-PD2 cells. (b) Colony formation of HeLa and PD45-Cd3-PD2 cells by soft agar assay.

Both morphology and PD evidences clearly indicated that chitosan had an important role in the reversal of aging-related process. It is known cellular senescence is controlled by the p53 and pRB tumor suppressor pathways [11,16,47]. To clarify whether the chitosan effect was involved in these pathways, the levels of p21, p53, pRB, and p16 expression in PD20, PD50, and PD48-Cd3-PD2 cells were compared. Fig 5a shows all senescence-associated proteins were higher in PD50 cells than PD20 cells, but no difference between PD50 cells and PD48-Cd3-PD2 cells, indicating chitosan-induced lifespan extending did not downregulate the proteins which are involved in tumor suppressor pathways. Furthermore, the soft agar assay showed that the chitosan-treated cells did not form colonies while the control HeLa cells formed colonies in soft agar (Fig 5b). These results suggested that the chitosan treatment could improve the proliferation capability of fibroblasts, but did not promote malignant transformation and induce cellular canceration.

### Effect of chitosan treatment on different cell population

**Fig 6:**
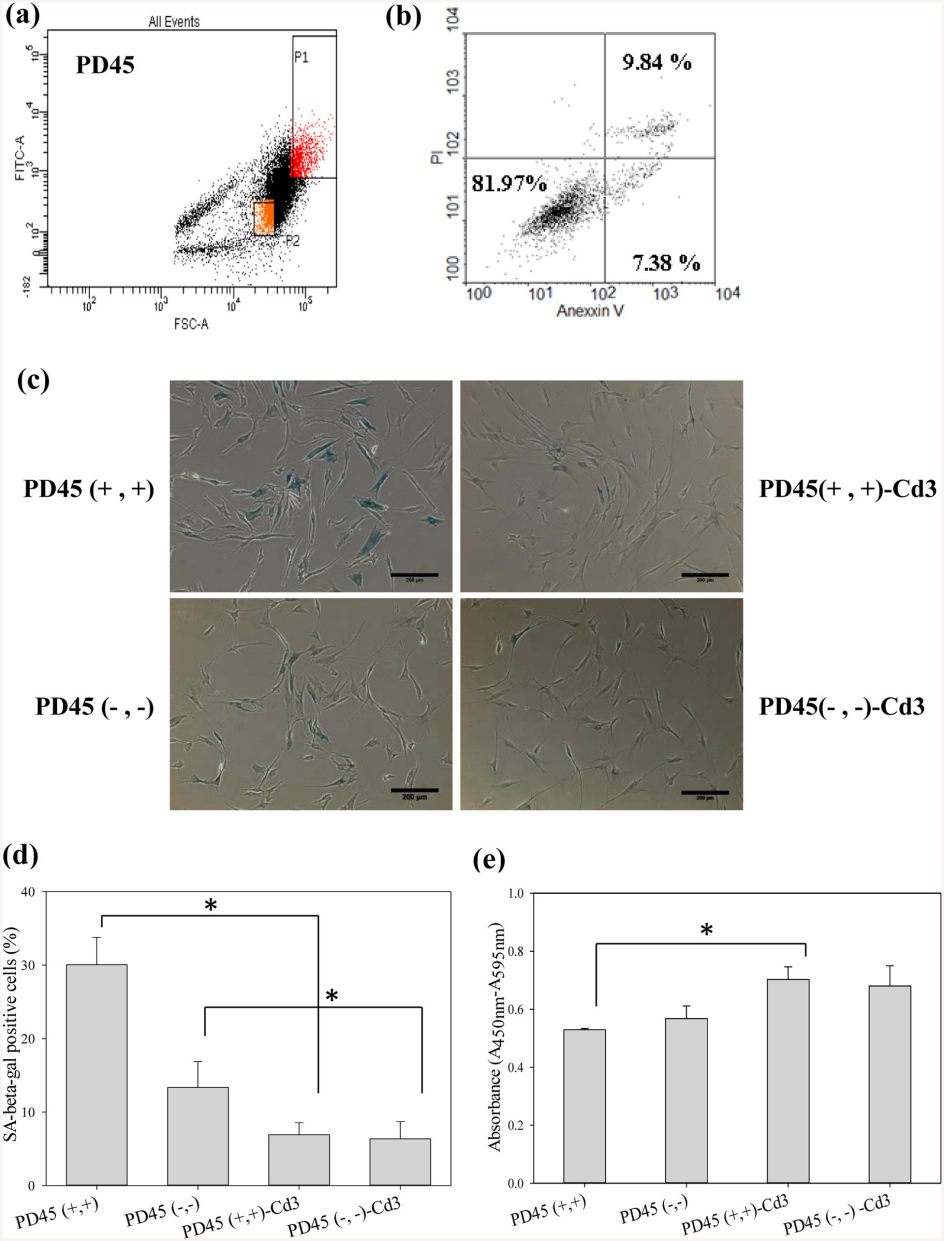
Fibroblasts sorted by FSC and FITC. (a) The gating range of cell sorting. P1: PD45 (+, +) population. P2: PD45 (-, -) population. (b) Viability of PD45 (+, +) cells determined by PI uptake and annexin-V-FITC labeling. (c) SA-β-gal staining of PD45 (+, +), PD45 (-, -), PD45 (+, +)-Cd3, and PD45 (-, -)-Cd3 cells. Scale bar = 200 μm (d) The percentage of PD45 (+, +), PD45 (-, -), PD45 (+, +)-Cd3, and PD45 (-, -)-Cd3 cells stained for SA-β-gal. At least 400 cells were calculated from ten randomly selected fields for each case. **p<0.01. (e) BrdU incorporation assay of PD45 (+, +), PD45 (-, -), PD45 (+, +)-Cd3, and PD45 (-, -)-Cd3 cells (n = 4). *p<0.05.

FSC, proportional to cell size, provides a suitable method of detecting cells greater than a given size in flow cytometry. In addition to cell size, autofluorescence is also a valid marker for cellular senescence *in vitro* [48]. To decide whether chitosan treatment extended fibroblast lifespan by increasing apoptosis or necrosis of older cell population to make average living cells more youthful, flow cytometry was used to sort the older and younger subpopulation of PD45 cells with FSC and FITC double-positive expression (denoted PD45 (+, +)) and double-negative expression (PD45 (-, -)), respectively (Fig 6a). After sorting, Fig 6b shows more than 80% of PD45 (+, +) cells were viable. Thus, PD45 (+, +) cells were appropriate for subsequent cultures but they indeed were older than PD45 (-, -) cells. As shown in Fig 6c–6e, the younger PD45 (-, -) cells exhibited lower SA β-gal-positive percentage and higher BrdU proliferation rate than the older PD45 (+, +) cells did. However, the two chitosan-treated cell populations (PD45 (+, +)-Cd3 and PD45 (-, -)-Cd3 cells) could reduce the ratio of SA β-gal-positive cells and increase the expression of BrdU labeling to similar level. Thus, chitosan treatment extended fibroblast lifespan possibly by increasing all cell proliferation, not by eliminating older cell population.

### Senescence-related expression of cells within the spheroids

**Fig 7:**
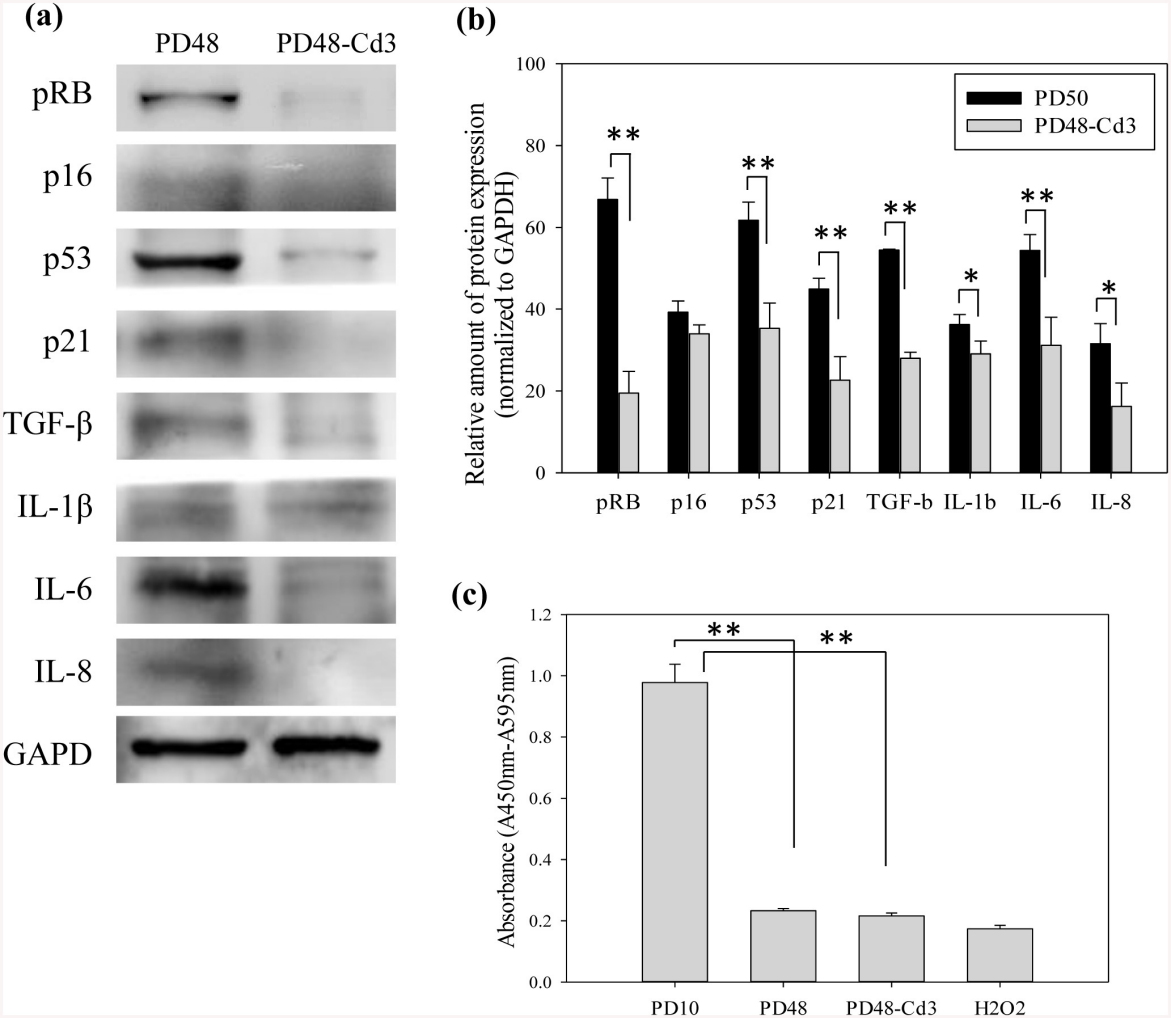
The characteristics of fibroblast spheroids after chitosan treatment. (a) Western blot results of proteins pRB, p16, p21, p53, TGF-β, IL-1β, IL-6 and IL-8 expression in PD48 and PD48-Cd3 cells. (b) The relative amount of protein pRB, p16, p21, p53, TGF-β, IL-1β, IL-6 and IL-8 expression in PD48 and PD48-Cd3 cells. (*p<0.05 and **p<0.01, n = 5). (c) BrdU incorporation assay of PD10, PD48, PD48-Cd3, and H_2_O_2_-treated cells (**p<0.01, n = 5).

Finally, to confirm whether the chitosan effect was involved in senescence-related pathways, the expression of senescence-associated molecular markers and SASPs in PD48-Cd3 spheroids was directly evaluated without further serial passages on TCPS. Clearly, cells in PD48-Cd3 spheroids showed significantly lower expression of senescence-associated molecular markers pRB, p16, p21 and p53, and SASPs TGF-β, IL-1β, IL-6 and IL-8 than PD48 cells did (*p<0.05 and **p<0.01, Fig 7a and 7b). Compared with Fig 5a, the low senescence-related protein expression was not maintained when chitosan-treated cells were reseeded on TCPS for further serial passages. Moreover, proliferation of PD10, PD48, PD48-Cd3 and H_2_O_2_-treated cells were compared. BrdU incorporation assay showed, in addition to PD10 cells, PD48, PD48-Cd3 and H_2_O_2_-treated cells expressed low absorbance (**p<0.01, Fig 7c). These results indicated that cells within CD48-Cd3 spheroids didn’t proliferate as highly as PD10 cells.

## Discussion

It is known biomaterials play critical roles in cell adhesion, migration, proliferation and function. However, the effect of biomaterials on cellular aging is not extensively investigated. Lou et al. demonstrated that the ethylene content of poly (ethylene-co-vinyl alcohol) could affect the senescence program of the cultured fibroblasts [36]. Thus, cells can interact with the underlying substrate, and the cellular aging process can be regulated due to the biomaterial characteristics. In this study, we tried to investigate whether cellular aging process could be regulated by changing the culture substrate from TCPS to chitosan. Fig 1 shows changes in cell morphology, decreased cell proliferation and increased SA β-gal activity were obvious when fibroblasts progressively encountered senescence on TCPS. Fig 2 shows senescent fibroblasts could suspend above chitosan to form multicellular spheroids with cell viability more than 85% after 3 days of incubation. Compared without chitosan-treated fibroblasts, chitosan-treated ones could decrease the percentage of SA β-gal-positive cells and undergo approximately 20–25 additional doublings (Fig 3). These results successfully assure that chitosan can delay the induction of cellular senescence to extend fibroblast lifespan. In addition, increased cellular motility was found in chitosan-treated cells by a scrape wound assay (Fig 4).

Anchorage-dependent fibroblasts require attachment to a substrate to proliferate [49,50], so fibroblasts should undergo programmed cell death when they suspended and aggregated on chitosan. Based on the live/dead assay [51] and PI/annexin-V-FITC labeling [52,53], the percentage of viable cells within the PD48-Cd3 spheroids was higher than PD48-Cd7 ones (Fig 2). One of possible reasons is that PD48-Cd7 cells were grown in suspension without anchorage attachment too long. The other possible reason is that cells within the spheroids could be regarded a stringent environment so it was more difficult for them to access enough oxygen and nutrition [54]. Therefore, the 3-day chitosan treatment provided cells an opportunity to change their fate before cell apoptosis or necrosis.

Cheng et al. reported that adipose-derived stem cells (ASCs) spontaneously formed spheroids on chitosan [44]. ASCs were induced to upregulate pluripotency marker genes to increase transdifferentiation efficiency into neurons and hepatocytes. Dissimilar to ASCs, fibroblasts do not possess the ability to undergo differentiation, and this may be that chitosan treatment enhanced the fibroblast proliferation instead of the differentiation-related properties. Overall, these results suggest chitosan not only can enhance the stemness and differentiation capabilities of stem cells but also can delay the induction of senescence in fibroblasts. Although the mechanisms of these functionalities are not clear, chitosan can be used to suspend and aggregate different cells with different fate determination.

It is known both p53 and pRB tumor suppressor pathways play important roles in the establishment and maintenance of cellular senescence [10,11]. Previous studies have reported that the inactivation of p21 or p53 and p16 or pRB with the interference of the viral proteins can cause a significant extension of *in vitro* lifespan [55–57]. Both p53/p21/pRB and p16/pRB pathways are responsible for arresting the senescence growth, but there are some differences between them. Usually, the p53/p21/pRB pathway is considered reversible, and inactivated p53 can extend the replicative lifespan of many human cells. Conversely, p16/pRB pathway is called irreversible growth arrest. Once the pRB is induced by p16, the growth arrest cannot be paused by inactivating or silencing any genes or proteins. In this study, fibroblasts during aging were demonstrated to display a reduction in the protein expressions of senescence-associated molecular markers after being treated with chitosan for 3 days without using viral proteins (Fig 7). The protein expression of p53, p21, and pRB of chitosan-treated cells was significantly decreased (p<0.01), but the protein expression of p16 was only decreased slightly. Thus, the p53/p21/pRB pathway was involved in the mechanism of chitosan-induced lifespan extension to delay the aging process.

Recently, SASPs were considered as important factors for cells to alter their microenvironment to induce the immune reaction. However, SASPs also would cause epithelial-to-mesenchyme transition to enhance tumorigenesis and metastasis [58–60]. In this study, all the expressions of TGF-β, IL-1β, IL-6, and IL-8 of fibroblasts were decreased evidently after treated with chitosan for 3 days (Fig 7b). Therefore, the risk for fibroblasts to release SASPs to induce immune and oncogenic reactions after chitosan treatment was appropriately decreased.

However, short-term chitosan treatment could not result in reduced expression of all senescence-associated proteins after cells reseeded on TCPS for serial passages (Fig 5a), so chitosan treatment-mediated lifespan extension did not continually downregulate p53, p21, and pRB expression, indicating that the treatment did not bypass the cellular aging process. Since p53 and pRB are regarded as tumor suppressor proteins to act as a barrier to tumorigenesis [11,16,47], and the soft agar assay proved that chitosan-treated cells did not form colonies (Fig 5b), chitosan treatment does not increase the risk of tumorigenesis for fibroblasts.

In the stringent condition, cells also might be killed by apoptosis or necrosis. Since older cells are more easily weeded out through competition with younger cells, average cell population became more youthful during the cell spheroid culture process. In this study, chitosan treatment could reduce the ratio of SA β-gal-positive cells and increase the expression of BrdU labeling of both older and younger subpopulation of PD45 cells to similar level (Fig 6). The older cells could maintain appropriate viability without considerable apoptosis or necrosis. In addition, Fig 2 shows most of cells in PD48-Cd3 spheroids were viable. Therefore, chitosan treatment extended fibroblast lifespan primarily by increasing all cell proliferation, not by eliminating older cell population.

Finally, the growth-stopping PD58 fibroblasts were tried to be cultured on chitosan to form cell spheroids. However, it was found that the short-term chitosan treatment could not stimulate the cells to divide again (S1 Fig). Since PD58 fibroblasts have entered a non-replicative state, chitosan treatment exerted better protection in pre-senescent fibroblasts. Therefore, once cells become irreversibly arrested, chitosan treatment cannot reverse the aging process to extend lifespan.

## Conclusions

In summary, chitosan could suspend human foreskin fibroblasts to significantly delay the appearance of replicative senescence. From the tissue engineering standpoint, delaying the induction of senescence may be useful to maintain cells in a younger state. Further investigation is required to clarify how chitosan activates the anti-senescence program.

## Supporting Information

S1 Fig The PD curve of cells with chitosan treatment at different PD. Red arrows indicate PD48 and PD58 cells were seeded on chitosan for 3 days and reseeded on TCPS for serial passages.
(TIF)
