# Supplementary figures and images for: Chitosan Treatment Delays the Induction of Senescence in Human Foreskin Fibroblast Strains
Source: PLoS One. 2015 Oct 14;10(10):e0140747. doi: 10.1371/journal.pone.0140747 (PMC4605659; doi:10.1371/journal.pone.0140747)

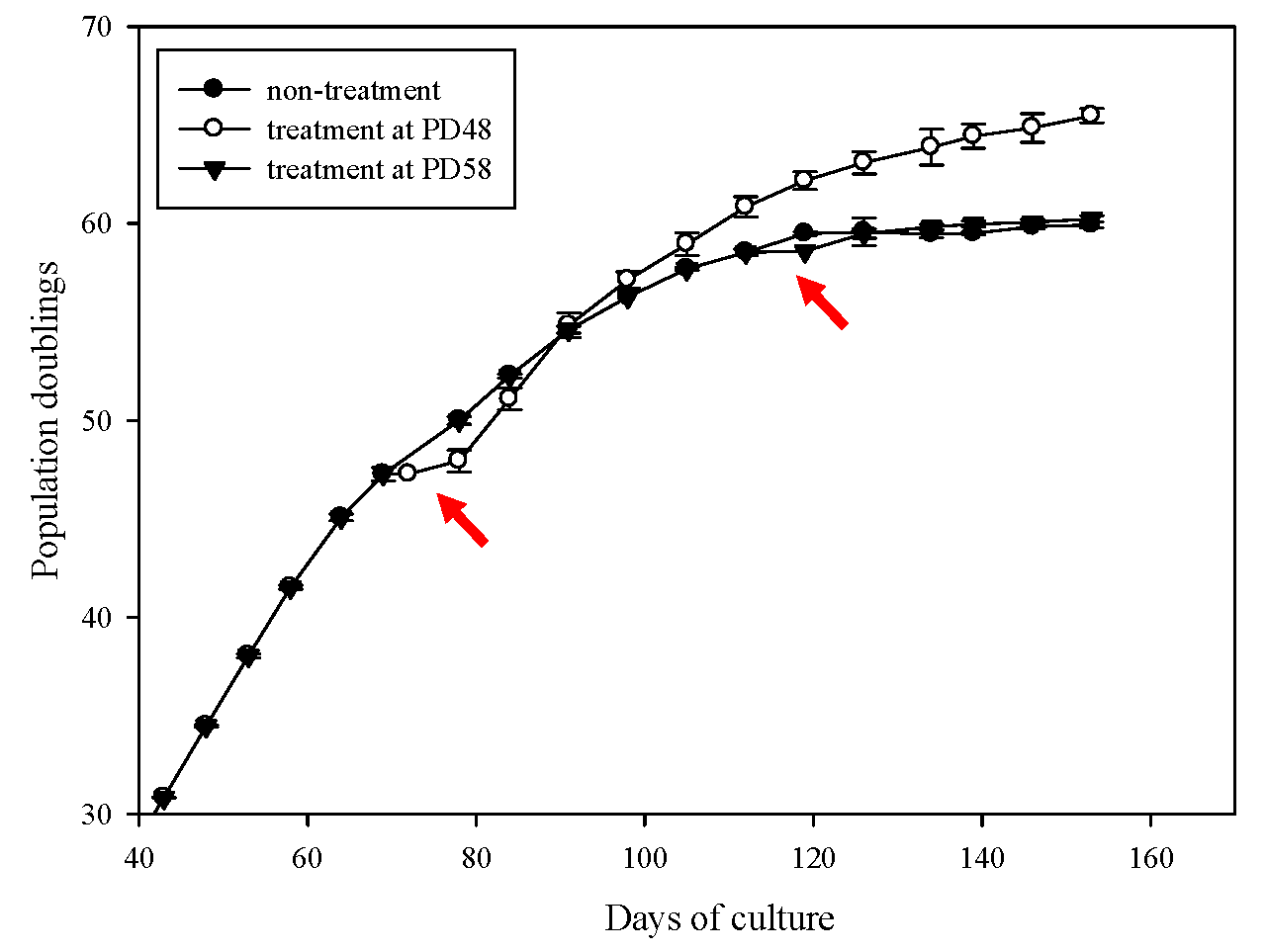

Supplement: S1 Fig — Red arrows indicate PD48 and PD58 cells were seeded on chitosan for 3 days and reseeded on TCPS for serial passages. (TIF) [file pone.0140747.s001.tif]
